# Expanding the phenotype of *PIGS*‐associated early onset epileptic developmental encephalopathy

**DOI:** 10.1111/epi.16801

**Published:** 2021-01-07

**Authors:** Stephanie Efthymiou, Marina Dutra‐Clarke, Reza Maroofian, Rauan Kaiyrzhanov, Marcello Scala, Javeria Reza Alvi, Tipu Sultan, Marilena Christoforou, Thi Tuyet Mai Nguyen, Kshitij Mankad, Barbara Vona, Aboulfazl Rad, Pasquale Striano, Vincenzo Salpietro, Maria J. Guillen Sacoto, Maha S. Zaki, Joseph G. Gleeson, Philippe M. Campeau, Bianca E. Russell, Henry Houlden

**Affiliations:** ^1^ Department of Neuromuscular Disorders UCL Queen Square Institute of Neurology University College London London UK; ^2^ Department of Pediatrics Division of Genetics University of California, Los Angeles Los Angeles CA USA; ^3^ Pediatric Neurology and Muscular Diseases Unit IRCCS Giannina Gaslini Institute Genoa Italy; ^4^ Department of Neurosciences, Rehabilitation, Ophthalmology, Genetics, Maternal and Child Health University of Genoa Genoa Italy; ^5^ Department of Pediatric Neurology Institute of Child Health Children’s Hospital Lahore Lahore Pakistan; ^6^ Research Center Saint Justine University Hospital Center University of Montreal Montreal QC Canada; ^7^ Department of Neuroradiology Great Ormond Street Hospital for Children London UK; ^8^ Department of Otolaryngology‐Head and Neck Surgery Tübingen Hearing Research Center Eberhard Karls University Tübingen Germany; ^9^ GeneDx Gaithersburg MA USA; ^10^ Clinical Genetics Department Human Genetics and Genome Research Division National Research Center Cairo Egypt; ^11^ Department of Neuroscience Rady Children's Institute for Genomic Medicine Howard Hughes Medical Institute University of California, San Diego San Diego CA USA

**Keywords:** congenital disorders of glycosylation, epilepsy, epileptic encephalopathy, exome sequencing, inherited GPI deficiency, neurodevelopmental disorders, phosphatidylinositol glycan class S, *PIGS*

## Abstract

The phosphatidylinositol glycan anchor biosynthesis class S protein (*PIGS)* gene has recently been implicated in a novel congenital disorder of glycosylation resulting in autosomal recessive inherited glycosylphosphatidylinositol‐anchored protein (GPI‐AP) deficiency. Previous studies described seven patients with biallelic variants in the *PIGS* gene, of whom two presented with fetal akinesia and five with global developmental delay and epileptic developmental encephalopathy. We present the molecular and clinical characteristics of six additional individuals from five families with unreported variants in *PIGS*. All individuals presented with hypotonia, severe global developmental delay, microcephaly, intractable early infantile epilepsy, and structural brain abnormalities. Additional findings include vision impairment, hearing loss, renal malformation, and hypotonic facial appearances with minor dysmorphic features but without a distinctive facial gestalt. Four individuals died due to neurologic complications. GPI anchoring studies performed on one individual revealed a significant decrease in GPI‐APs. We confirm that biallelic variants in *PIGS* cause vitamin pyridoxine‐responsive epilepsy due to inherited GPI deficiency and expand the genotype and phenotype of *PIGS*‐related disorder. Further delineation of the molecular spectrum of *PIGS*‐related disorders would improve management, help develop treatments, and encourage the expansion of diagnostic genetic testing to include this gene as a potential cause of neurodevelopmental disorders and epilepsy.

## 1 INTRODUCTION

Congenital disorders of glycosylation (CDGs) are a rapidly growing heterogeneous group of genetic conditions. Inherited glycosylphosphatidylinositol‐anchored protein (GPI‐AP) deficiencies (IGDs) are a subset of CDGs that account for 0.15% of all neurodevelopmental disorders.^1^ In many cases, IGDs result from the failure of the GPI anchor to regulate APs on the external cell surface. This has consequences for early human neurogenesis and neurodevelopment.^2^, ^3^, ^4^, ^5^ To date, 17 known IGDs lead to a wide range of symptoms, including variable intellectual disabilities and developmental impairment, seizures, hypotonia, weakness, ataxia, congenital malformations, and dysmorphic facial features. There are more than 150 human GPI‐APs, which often play a role in synaptic function, development of the central nervous system (CNS), and plasticity; defects in several of these proteins have been implicated in various neurological disorders.^2^

Developmental and epileptic encephalopathies include a range of severe epilepsies in which intractable seizures are accompanied by impairment of motor and cognitive functions.^6^ Recently, many cases of IGD have been found among individuals with intellectual disability and intractable seizures. Early infantile epileptic encephalopathy‐55 (EIEE; Mendelian Inheritance in Man [MIM] #617599), a severe form of epilepsy with frequent tonic seizures or spasms beginning in infancy, and developmental delay or regression, has been linked to several genes important for GPI biosynthesis and attachment. For example, *PIGB*, *PIGQ*, *PIGP*, and *PGAP1* have all been linked to novel autosomal recessive IGDs^7^, ^8^, ^9^; *PIGA* is an X‐linked recessive disorder.^10^

Phosphatidylinositol glycan class S (*PIGS*; MIM #610271) encodes a GPI‐AP, specifically a subunit of the GPI transamidase complex that catalyzes the attachment of preformed GPI to proteins containing a C‐terminal GPI attachment signal. Biallelic variants in *PIGS* have recently been implicated in human diseases, with the observation of seven individuals affected with EIEE.^11^, ^12^ Phenotypes included severe global developmental delay, seizures, hypotonia, weakness, ataxia, and dysmorphic facial features, but also multiple joint contractures (consistent with fetal akinesia) in two fetuses. All seven individuals showed a GPI‐AP deficiency profile. Notably, one individual showed a reduction in seizure frequency with pyridoxine (100 mg per day).

We describe six individuals (from five unrelated families) carrying biallelic variants in *PIGS* and presenting with hypotonia, severe global developmental delay, infantile onset (intractable) seizures, and different CNS anomalies identified on brain imaging. Two patients in this cohort are responsive to pyridoxine. This study provides additional evidence for *PIGS* as an emerging gene associated with autosomal recessive EIEE and describe the clinical characteristics, prognosis, and treatment outcomes, to improve patient care.

## 2 MATERIALS AND METHODS

### 2.1 Human participants

The study was approved by the ethics institutional review board of University College London and additional local ethics committees of the participating centers (see also Supplementary Data S1). Informed consent was obtained from all families.

### 2.2 Exome and Sanger sequencing

Biallelic variants in the *PIGS* gene were identified by exome sequencing (ES) as previously described.^13^, ^14^ All variants are listed using canonical transcript NM_033198.4.

### 2.3 Computational and in vitro splice analysis

Computational analysis of the c.174G>C variant was performed as previously described.^15^ To assess the predicted splicing impact of the c.174G>C (p.Gln58His) variant in Patients 1 and 2, an in vitro splicing assay was performed with minor modifications.^16^

### 2.4 Flow cytometry analyses

Fluorescence‐activated cell sorting analysis to assess for reduction in cell‐surface expression of GPI‐APs was done for Patient 6 and performed as previously described.^15^

## 3 RESULTS

### 3.1 Clinical findings

**FIGURE 1:**
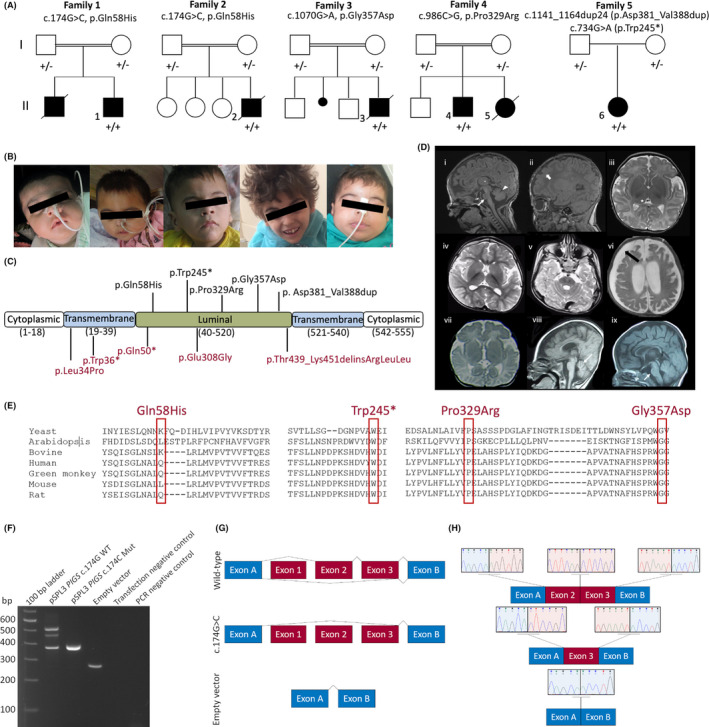
Clinicogenetic, radiological, and molecular findings of individuals in our cohort. (A) Pedigrees and segregation results (+ represents the variant) of the five families carrying biallelic *PIGS* variants. (B) Patients 1–5 show coarse facial features, including arched eyebrows, almond‐shaped palpebral fissures, depressed nasal bridge, deep philtrum, and pointed chin. (C) A schematic representation of the PIGS protein showing the position of all *PIGS* variants identified, with previously reported variants in red. (D) Magnetic resonance imaging findings of (i–iii) Patient 6 at 5 weeks. Sagittal T‐weighted image at the midline (i) shows small pons (arrow), early superior cerebellar vermian atrophy (arrowhead), and a large massa intermedia (star). Parasagittal T1‐weighted image (ii) shows underdeveloped and undersulcated frontal lobes (arrow). Compare the sulcation in the anterior aspects with the posterior cerebral hemisphere. Axial T2‐weighted image (iii) shows underopercularization due to underdevelopment of the frontal and anterior temporal lobes and large thalami (star). (iv, v) Patient 4 at 5 years. Axial T2‐weighted images at the supratentorial (iv) and infratentorial (v) levels show generalized brain atrophy and delayed maturation of myelin. Note again the worse atrophy anteriorly in the frontotemporal regions. (vi) Patient 5 at 5 months. Axial T2‐weighted image shows atrophy‐related bilateral subdural hygromas (arrow). (vii) Patient 1 at 3.5 months. Axial T2‐weighted image shows underopercularization bilaterally secondary to frontotemporal atrophy. Frontal dysgyria is also noted. (viii) Patient 2 at 34 weeks. Sagittal T1‐weighted image shows microcephaly, with greater volume reduction in the frontal lobe, as well as early superior cerebellar vermian atrophy (arrow). (ix) Patient 3 at 14 months. Sagittal T1‐weighted image shows a small pons and globally reduced cerebral white matter volume (worse anteriorly) associated with hypoplasia of the corpus callosum. (E) Interspecies alignment performed with Clustal Omega shows the complete conservation down to invertebrates of the amino acid residues affected by the substitutions. (F) RNA analysis of the *PIGS* c.174G>C variant. Gel electrophoresis of the reverse transcriptase polymerase chain reaction (PCR) wild‐type (WT), mutant (Mut) c.174G>C variant, and empty pSPL3 vector amplicons. Transfection negative and PCR negative controls performed as expected. (G) Overview of the splicing of the wild‐type (upper panel), patient (middle panel), and empty vector (lower panel) splicing. Note that although exon 1 was cloned into the vector, it was not spliced to exon A and exon 2, as it lacks a splice acceptor site. (H) Visualization of the exon junction sequence of the wild‐type (upper panel), patient (middle panel), and empty vector (lower panel)

We identified six novel patients from five families with biallelic variants in the *PIGS* gene found by trio ES. Parental consanguinity was reported in four of the five families, all of whom had homozygous variants in *PIGS*. The clinical features of the affected individuals are summarized in Table S1 and are described further in Supplementary Data S1. All individuals presented with severe global developmental delay, intractable early infantile onset seizures, and absent speech. Patients 2, 3, and 5 died from complications of recurrent respiratory infections. Intractable seizures requiring multiple antiepileptic drugs (AEDs) were noted in all patients. The electroencephalographic (EEG) features in our cohort were variable, influenced by the age of the patients and the effect of the specific variant; however, all subjects showed EEG recording indicative of a severe developmental epileptic encephalopathy, that is, pseudohypsarrhythmia with runs of high amplitude and slow waves predominant over posterior regions associated with multifocal epileptic abnormalities within a diffusely slowed high amplitude during wakefulness and sleep or within a burst‐suppression pattern. All of the individuals in this cohort had abnormal findings on brain magnetic resonance imaging, including atrophy of the frontal and anterior temporal lobes, with or without generalized brain atrophy. Frontal atrophy led to the development of bilateral subdural hygromas in one patient (Patient 5). We noted hypoplasia of the pons in four patients. One individual with severe pontine hypoplasia had large thalami and massa intermedia. All six affected individuals had microcephaly (−3 SD). Behavioral anomalies in two siblings (Patients 4 and 5) included autistic features and excessive laughing/crying. Other variable features include renal malformation (1/6, 17%), visual impairment (3/6, 50%), severe hearing loss (2/6, 33%), and acquired arthrogryposis (2/6, 33%). Facial dysmorphism was observed in most of the affected individuals (Figure 1B), mainly consisting of coarse features such as thick arched eyebrows, almond‐shaped palpebral fissures, depressed nasal bridge, and deep philtrum. Brachytelephalangy was also observed in three subjects (Patients 1–3), although in one of them (Patient 1) there was no evident epiphyseal or metaphyseal abnormality upon skeletal survey. Alkaline phosphatase levels (normal value = 0–281 U/L) were normal in Patient 2 (206 U/L) and mildly elevated in Patient 4 (445 U/L). This finding was not available for other affected individuals.

Although seizure management with AEDs is challenging, Patients 1 and 6 improved with pyridoxine (15 mg/kg/day) and folinic acid (1 mg/kg/day). Patient 1 reported decrease in seizure frequency, and Patient 6 reported improved developmental progress.

### 3.2 Genetic findings

ES in the probands of Families 1 and 2 revealed a novel homozygous variant in exon 2 of *PIGS*, c.174G>C (p.Gln58His), predicted to affect RNA splicing. The variant segregated with the disease in both families (Figure 1A) and was located within a 14‐Mb region of homozygosity. The variant was absent in publicly available population databases, including gnomAD, Exome Sequencing Project, GME Variome, and Iranome, as well as in more than 14,000 in‐house exomes. In Family 3, a novel homozygous missense variant c.1070G>A (p.Gly357Asp) within a 10‐Mb block of homozygosity was detected. The variant occurs at a highly conserved amino acid residue (Figure 1E), has a Combined Annotation Dependent Depletion score of 27 and Genomic Evolutionary Rate Profiling (GERP) score of 5.49, and segregated with the disease in the family. A novel segregating homozygous missense variant c.986C>G, (p.Pro329Arg), altering a highly conserved amino acid (Figure 1E), was identified for Family 4 (GERP score = 5.37). Both p.Gly357Asp and p.Pro329Arg are predicted to have a deleterious impact on the resulting PIGS protein, based on in silico predictions (Table S2).

In Family 5, Patient 6 has two variants. One variant is a paternally inherited in‐frame insertion of eight amino acids in a nonrepeat region, as c.1141_1164dup24 (p.Asp381_Val388dup) in exon 10. This variant has been previously reported in the literature in a male of Chinese descent with *PIGS*‐related disorder and seen in gnomAD in a heterozygous carrier state in five individuals of East Asian ethnicity. The second variant is a maternally inherited nonsense variant c.734G>A (p.Trp245*) in exon 7. This variant has been seen in gnomAD but only in the heterozygous carrier state in one individual of African ethnicity. Table S2 shows the allele frequency and American College of Medical Genetics and Genomics criteria‐based assessment of these variants.

### 3.3 Splicing analysis

Computational splice analysis of the c.174G>C variant predicted two possible effects that prompted RNA studies: (1) a decrease in the splice donor scores between 12.6% and 43.3% and (2) the abolishment of an exonic splicing enhancer motif (Figure S2). In vitro RNA analysis of the wild‐type c.174G disclosed a 508‐bp amplicon including exons 2 and 3, as well as a 369‐bp amplicon with only exon 3 (Figure 1F,G). This likely reflects alternative splicing (NM_033198.4/ENST00000308360.8 and ENST00000395346.6). The amplicon with the homozygous c.174G>C variant revealed only exon 3 (369 bp), indicating a skipping of exon 2 (Figure 1F,G) that was Sanger sequence confirmed (Figure 1H). This out‐of‐frame deletion of exon 2 (r.35_174del) would result in a premature stop codon (p.Glu12Alafs*31).

### 3.4 Flow cytometric analysis

**FIGURE 2:**
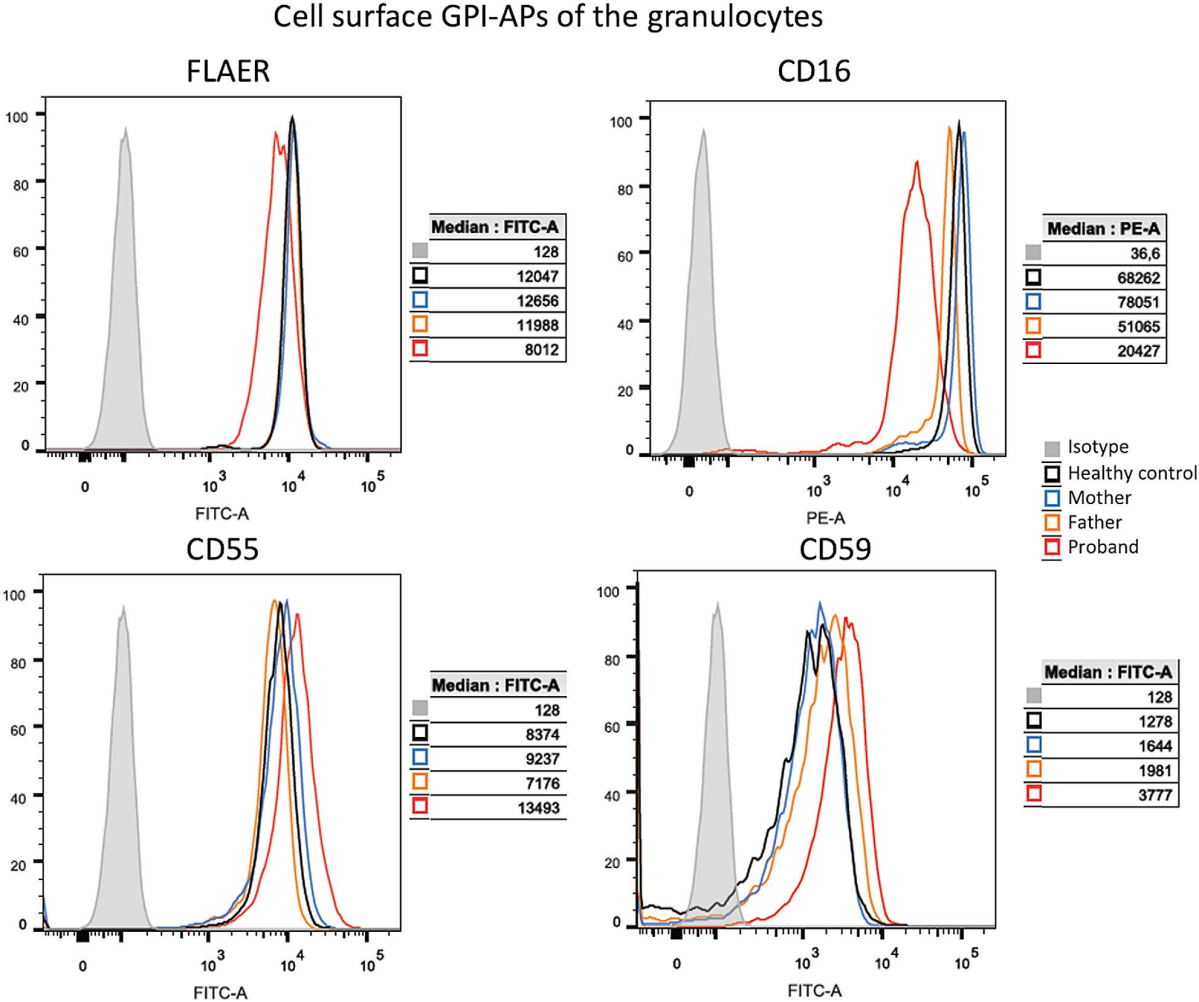
Impact of the *PIGS* variants on individual granulocyte cell‐surface glycosylphosphatidylinositol‐anchored protein (GPI‐AP). Fluorescence‐activated cell sorting (FACS) analysis on granulocytes indicated that individual red blood cells from Patient 6 of Family 5 had less signal of CD16, CD55, and CD59 than age‐matched control cells, showing a reduced cell‐surface expression. FLAER, fluorescent aerolysin; FITC, fluorescein isothiocyanate; PE, phycoerythrin

Individual 6 had significantly lower levels of CD16 and fluorescent aerolysin (FLAER) in granulocytes, the most sensitive markers of inherited GPI deficiency (Figure 2). There was no decrease for CD55 and CD59 in granulocytes, but these are less sensitive markers of inherited GPI deficiency. There was also low CD14 in monocytes and low FLAER in lymphocytes (Figure S3). This is consistent with the previously reported case series that showed biallelic variants in *PIGS* lead to reduced amounts of GPI‐AP at the cell surface.^15,16^ Due to the country of origin of the two patients still alive and the limited clinical facilities, we could not replicate the flow cytometric analysis in additional individuals.

## 4 DISCUSSION

Our study expands the phenotypic and genotypic spectrum of *PIGS* variants, which have been previously associated with profound neurodevelopmental impairment, EIEE, and premature death. We expand the known population of seven individuals with biallelic pathogenic variants in *PIGS* reported so far by adding five novel patients. We report five variants, four of which were previously unreported; namely, one truncating, one in‐frame duplication, and three missense variants. According to gnomAD constraint scores, *PIGS* is intolerant toward both missense variants (*Z* = .7801) and biallelic loss of function variants (probability of being loss‐of‐function intolerant = 0, pREC = .9943).

All patients had hypotonia, microcephaly, global developmental delay, absent speech, and infantile onset epilepsy. Brachytelephalangy was also observed in three patients. Although first reported in *PIGV*‐related disorders,^17^ it is not present in all GPI deficiencies.^11^ We also identified neuroimaging findings in our patients, consistent with previously published studies that described cerebellar and diffuse cortical atrophy in individuals carrying biallelic *PIGS* variants.^11^, ^12^ In this cohort, vision impairment, hearing loss, and behavioral abnormalities were also commonly observed. Taken together, biallelic (gene‐disruptive) variants in *PIGS* are associated with an early infantile encephalopathy refractory to standard AEDs co‐occurring with global developmental delay, severe hypotonia, and visual and hearing impairment.

Elevated alkaline phosphatase levels have been observed in other disorders of GPI anchor synthesis.^7^ In our cohort, only one of two tested patients showed mildly elevated alkaline phosphatase levels, supporting the evidence that increased alkaline phosphatase levels are not observed in all GPI deficiencies.^11^

GPI‐AP function is also implicated in vitamin B6 and folate transport, nucleotide metabolism, and lipid homeostasis. B6‐dependent enzymes are involved in the metabolism of amino acids, neurotransmitters, molybdenum cofactor, and sphingolipids.^18^ Two of our patients were treated with pyridoxine and folinic acid, with some benefit in seizure control even before the genetic analysis; moreover, we could not measure the cerebrospinal fluid pyridoxal‐5‐phosphate levels primarily due to the limited testing ability of their referring hospitals. Although anecdotal, previous studies have also shown the effects of pyridoxine in other IGDs.^19^ Pyridoxine has been shown effective in various GPI deficiency disorders, although there is no clear evidence describing the effect of folinic acid. The ketogenic diet has also been previously reported to improve outcome in cases of *PIGA* deficiency and may be a consideration for individuals with *PIGS*‐related disorder.^20^

This study further reviewed the clinical features of *PIGS*‐related disorders. Distinguishing *PIGS*‐related disorders from other pediatric neurological syndromes will rely on the combination of physical examination findings and identifying the phenotypic features resulting from disruption of *PIGS*. These include intractable epilepsy, severe developmental delay, hypotonia, and coarse faces. Our data further highlight the importance of including the screening of the *PIGS* gene in the case of infantile epilepsy and may serve for improved interpretation of new *PIGS* variants with the help of biochemical findings and flow cytometry analysis. Finally, *PIGS* is not found on most clinically utilized epilepsy gene panels, suggesting that it may be a more frequent cause of early infantile epilepsy than reported. As epilepsy genetic testing expands and ES becomes more available, more patients will likely be identified in the future.

## CONFLICT OF INTEREST

M.J.G.S. is an employee of GeneDx. None of the other authors has any conflict of interest to disclose.

ETHICAL PUBLICATION STATEMENT

We confirm that we have read the Journal's position on issues involved in ethical publication and affirm that this report is consistent with those guidelines.

## Supporting information

Table S1

Table S2

Supplementary Material
