# Supplementary material for: Expanding the phenotype of PIGS‐associated early onset epileptic developmental encephalopathy
Source: Epilepsia. 2021 Jan 7;62(2):e35–41. doi: 10.1111/epi.16801 (PMC7898547; doi:10.1111/epi.16801)
Supplement: Supplementary file 2 — Table S2 [file EPI-62-e35-s002.docx]

|  | **Patient ID**  **(Family)** | **Patients 1-2**  **(F1-II:1; F2-II:1)** | **Patient 3**  **(F3-II:1)** | **Patients 4-5**  **(F4-II:1;F4-II:2)** | **Patient 6**  **(F5-II:1)** | |
| --- | --- | --- | --- | --- | --- | --- |
| **Variant annotation** | GRCh37/hg19  position  (DNA change) | chr17:26898067 | chr17:26883855 | chr17:26883939 | chr17:26883200 | chr17:26887152 |
|  | cDNA change (NM_033198.4) | c.174G>C | c.1070G>A | c.986C>G | c.1141_1164dup24 | c.734G>A |
|  | Protein change | p.(Gln58His) | p.(Gly357Asp) | p.(Pro329Arg) | p.(Asp381_Val388dup) | p.(Trp245*) |
|  | Inheritance | Hom | Hom | Hom | Comp Het | Comp Het |
|  | dbSNP ID | - | - | - | rs769890071 | rs1249675321 |
|  | Variant seen in family | F1-F2 | F3 | F4 | F5 | F5 |
| **Allele frequencies (PM2)** | gnomAD v3  (highest subpopulation) | - | - | - | 0.029% (5/17248) | 0.0115% (1/8732) |
|  | gnomAD v2.1.1  (highest subpopulation) | - | - | - | - | 0.011% (1/8714) |
|  | Frequency in ensembl browser | - | - | - | - | - |
|  | Iranome | - | - | - | - | - |
|  | GME Variome | - | - | - | - | - |
|  | Frequency in in-house database‡ | - | - | - | - | - |
|  | Frequency in GeneDx database | 0.001% (1/139678), no homs | 0.001% (1/130874), no homs | - | 1/164434, no homs | 0.001% (1/164434), no homs |
| ***In silico* predictions (PP3)** | GERP | 5.05 | 5.49 | 5.37 | - | 5.84 |
|  | CADD | 25.2 | 27.4 | 28.9 | 22.3 | 38 |
|  | Polyphen-2 | B (0.431) | PD (1) | PD (0.964) | - | - |
|  | SIFT | T (0.09) | D (0) | D (0) | - | - |
|  | Provean | N (-1.725) | D (-6.756) | D (-6.029) | D (-11.319) | D (-14.144) |
|  | MutationTaster | DC (0.999) | DC (0.9999) | DC (0.9999) | - | DC (1) |
| **ACMG** | Overall classification | Likely pathogenic (PVS1, PS3 PM2, PM5, PP1, PP4) | VUS (PM2, PP3, PP4) | Likely pathogenic (PP1, PM2, PP3, PP4) | Likely pathogenic (PM3, PM4, PP3, PP4) | Pathogenic (PVS1, PM2, PP3, PP4) |
|  | Additional  supporting evidence: | Null variant: affecting the last base before splice site | - | - | - | Null variant: stop gain variant |

**Supplementary Table 2.** *PIGS* intragenic variants identified in our cohort.

Abbreviations: B = benign; CADD = Combined Annotation Dependent Depletion; Comp het = compound heterozygous; D = damaging; DC = disease causing; F = family; GERP = Genomic Evolutionary Rate Profiling; Hom = homozygous; homs = homozygous entries; N = neutral; PM = pathogenic moderate; PP = pathogenic supporting; PS = pathogenic strong; PVS = pathogenic very strong; SIFT = Sorting Intolerant From Tolerant; T = tolerated; VUS = variant of uncertain significance. ‡ Database of 16,000 control exomes.
