# Supplementary material for: Expanding the phenotype of PIGS‐associated early onset epileptic developmental encephalopathy
Source: Epilepsia. 2021 Jan 7;62(2):e35–41. doi: 10.1111/epi.16801 (PMC7898547; doi:10.1111/epi.16801)
Supplement: Supplementary file 3 — Supplementary Material [file EPI-62-e35-s003.docx]

**Expanding the phenotype of PIGS-associated early-onset epileptic developmental encephalopathy**

Stephanie Efthymiou^1*^, Marina Dutra-Clarke^2*^, Reza Maroofian^1^, Rauan Kaiyrzhanov^1^, Marcello Scala^3,4^, Javeria Reza Alvi^5^, Tipu Sultan^5^, Marilena Christoforou^1^, Thi Tuyet Mai Nguyen^6^, Kshitij Mankad^7^, Barbara Vona^8^, Aboulfazl Rad^8^, Pasquale Striano^3,4^, Vincenzo Salpietro^3,4^, Maria J. Guillen Sacoto^9^, Maha S. Zaki^10^, Joseph G. Gleeson^11^, Philippe M. Campeau^7^, Bianca E. Russell^2^, Henry Houlden^1^

**Supplementary methods**

**2.1 Human participants**

Family 5 was recruited through GeneMatcher (<https://genematcher.org/>). Clinical ascertainment included physical examination, medical history interviews, and specialized consultation by paediatric neurologists and clinical geneticists. Two independent neurologists reviewed brain MRIs and EEGs. GPI anchoring studies for Patient 6 was approved by the ethics Institutional Review Board of CHU-Sainte Justine and Université de Montréal.

**2.2 Exome and sanger sequencing**

ES was performed on genomic DNA extracted from venous blood or buccal samples according to standard procedures and performed as a trio with parental samples. For all the affected individuals, ES and data analysis was performed as described.**^18; 19; 26^** For Patient 6, ES was performed in the setting of routine diagnostic testing without the requirement for institutional ethics approval. The candidate variants were confirmed, and segregation analysis was performed by Sanger sequencing.

**2.3 Computational and *in vitro* splice analysis**

Briefly, a 1,535 bp genomic region including exons 1 to 3 was PCR amplified from the genomic DNA of a control individual and proband with primers containing *Xho*I and *Bam*HI restriction sites (primers available on request). PCR fragment ligation in a linearized pSPL3-vector preceded transformation into DH5α competent cells (NEB 5-alpha, New England Biolabs) and overnight incubation. The wild-type and mutant-containing vector sequences were confirmed by Sanger sequencing and transfected into HEK 293T cells (ATCC) with FuGENE 6 Transfection Reagent (Promega). Empty vector and transfection negative reaction controls were included. Total RNA was prepared from 24**-**hour post-transfected cells using a miRNAeasy Mini Kit (Qiagen) that was reverse transcribed using a High Capacity RNA-to-cDNA Kit (Applied Biosystems). Amplified fragments were visualized on a 1% agarose gel (Sigma) and Sanger sequenced. cDNA amplicons from the wild-type individual were cloned using the TA cloning dual promoter with pCRII kit (Invitrogen).

**Supplementary clinical data**

#### Family 1 (Patient 1)

#### This is a 3-year-old male born preterm at 7 months gestational age to consanguineous Pakistani parents. He presents with neonatal-onset multifocal clonic and myoclonic seizures, global developmental delay, impaired vision, sensorineural hearing loss, and stereotypic mouth twitching. Electroencephalography revealed pseudo-hypsarrhythmic pattern during wakefulness with runs of 2-3-Hz high amplitude and synchronous slow waves predominant over posterior regions associated with few spikes and slow-wave spikes over temporal regions (Supplementary Figure 1). His seizures have been managed by various AEDs, including levetiracetam, nitrazepam, and topiramate. Supplementation with pyridoxine (100 mg per day) reduced the frequency of seizures for the first two years. At 3 years old he is microcephalic at 43cm (-3.9SD) with no growth failure or malformations. Brain MRI done at 3.5 months of age showed under-opercularization bilaterally secondary to frontotemporal atrophy, as well as frontal dysgyria (Figure 1D). He had an affected brother who started to have focal-clonic seizures since the first month of life. He was developmentally delayed with no gaze fixation or neck holding. EEG was suggestive of epileptic encephalopathy and he died at 4 months of age due to respiratory infection.

#### Family 2 (Patient 2)

#### This male was born from consanguineous parents from Pakistan following an uneventful pregnancy. He did not cry at birth and later presented with neonatal multifocal clonic seizures, developmental delay, as well as vision and hearing impairment. His EEG at age 1.5 years revealed multifocal epileptic abnormalities within a diffusely slowed high-amplitude during wakefulness and sleep intermixed with a burst-suppression pattern, indicative of developmental epileptic encephalopathy (Supplementary Figure 1). He was treated with multiple antiseizure medications but not pyridoxine supplementation. He was microcephalic with no significant growth failure (unknown parameters) and behavioral abnormalities. Brain MRI done at 1 month of age revealed microcephaly, with greater volume reduction in the frontal lobe, as well as early superior cerebellar vermian atrophy (Figure 1D). He died at the age of 1 year 9 months status epilepticus.

#### Family 3 (Patient 3)

#### This is a male born after an uneventful pregnancy and delivery to consanguineous parents of Pakistani origin. He has developmental delay and febrile generalized tonic-clonic seizures. Seizures began at 2 months of age, and throughout infancy, multiple seizure types developed. He started with focal-clonic seizures which became multifocal by 8-10 months of life followed by tonic spasms in the late first year with progression to generalized tonic-clonic seizures in the second year of life. EEG at age 3.5 years showed multifocal epileptic abnormalities within a burst-suppression pattern (Supplementary Figure 1). He partially respond to a combination of levetiracetam, carbamazepine and valproic acid and he has not been treated with pyridoxine supplementation. He was non-verbal and microcephalic (unknown parameters) with no behavioral symptoms. His neuromuscular examination reveals spastic gait and truncal hypotonia. His brain MRI done at 14 months revealed a small pons and globally reduced cerebral white matter volume associated with hypoplasia of the corpus callosum (Figure 1D). He died at the age of 2 years due to respiratory failure following an acquired respiratory tract infection.

#### Family 4 (Patients 4 and 5)

#### Two affected siblings (Patient 4 and Patient 5) were born to consanguineous Egyptian parents after an unremarkable pregnancy. Patient 4 is an 8-year-old male with severe global developmental delay, neonatal-onset intractable tonic seizures (GTS), mild autistic features, and excessive laughing. From the age of 12 months, he suffered from recurrent seizures with focal onset followed by tonic-clonic seizures, triggered by fever. He has not been responsive to a combination of valproate, levetiracetam, topiramate, and clonazepam resulting in frequent admissions to the intensive care unit. At age 8, his growth parameters included weight at 16kg (-3.2SD) and height at 115cm (-22.5SD). He is microcephalic (46.5 cm (-4.2SD). He is non-ambulatory and non-verbal. He has mild hypotonia (able to sit and stand supported), brisk reflexes, and mild acquired arthrogryposis of the knees. Brain MRI shows generalised brain atrophy (worse anteriorly in the frontotemporal regions) and delayed maturation of myelin (Figure 1D). At age 6 years, his EEG showed diffusely slowed, low-voltage, monomorphic background activity indicative of subcortical -cortical dysregulation with no evidence of epileptic abnormalities (Supplementary Figure 1). The affected sister (Patient 5) similarly has a global developmental delay with neonatal onset of intractable focal seizures followed by secondary generalized myoclonic jerks associated with lip-smacking and eye blinking movements. Her seizures are not responsive to valproate, levetiracetam, topiramate and phenytoin. At 9 months of age, she is not alert, does not follow objects, had no acquired milestones, and is fed by nasogastric tube. Growth parameters at 9 months include weight at 7.7kg (1^st^ percentile) and height at 65cm (-22.8SD). She has microcephaly at 40cm (-3.2SD). She presents with excessive crying, hypotonia, and spasticity. Her brain MRI shows atrophy related bilateral subdural hygromas (Figure 1D). She died at the age of 1 due to intractable seizures after being ventilated for 2 weeks. Neither sibling was treated with pyridoxine supplementation.

#### Family 5 (Patient 6)

#### Patient 6 is a 2-year-old female born to non-consanguineous parents of Chinese descent. She presents with multifocal seizures at four weeks of life. EEGs showed frequent independent and synchronous spike-wave discharges over right posterior quadrant and occasionally at left posterior quadrant. Her seizures are not well controlled on multiple AEDs. Her current treatment includes clobazam, cannabidiol, diazepam, folinic acid, levetiracetam, perampanel, pyridoxine, zonisamide, and clonazepam. Despite treatment, at 24 months her seizures occur multiple times daily, often with rapid eye blinking and lip-smacking, consistent with myoclonic seizures. In addition to her seizures, she presents with global developmental delay, cortical visual impairment, and profound hypotonia. Though seizures have not been well controlled, parents report an increase in eye tracking, alertness, and fine motor skills after starting B6. Her development has slowly improved and at 16 months she has a social smile and is making vocalizations, though she does not babble or have any words. She rolls but cannot sit without support or support her head consistently. Growth parameters at 24 months include weight of 9.32 kg (-2.4SD), length of 83 cm (24^th^ percentile). She was normocephalic at birth then developed progressive microcephaly with head circumference of 43.6 cm (-2.7SD). She only eats pureed foods due to poor swallow coordination. Brain MRI showed small pons (Figure 1D, arrow), early superior cerebellar vermian atrophy (arrow head) and large massa intermedia (star), as well as underdeveloped and simplified frontal lobes (arrow) and opercularization due to underdevelopment of the frontal and anterior temporal lobes and large thalami (star). Following a febrile urinary tract infection, renal ultrasound identified asymmetric kidneys with her right kidney being 1.4 cm larger than the left and possible duplicated collecting system of the right kidney.

**
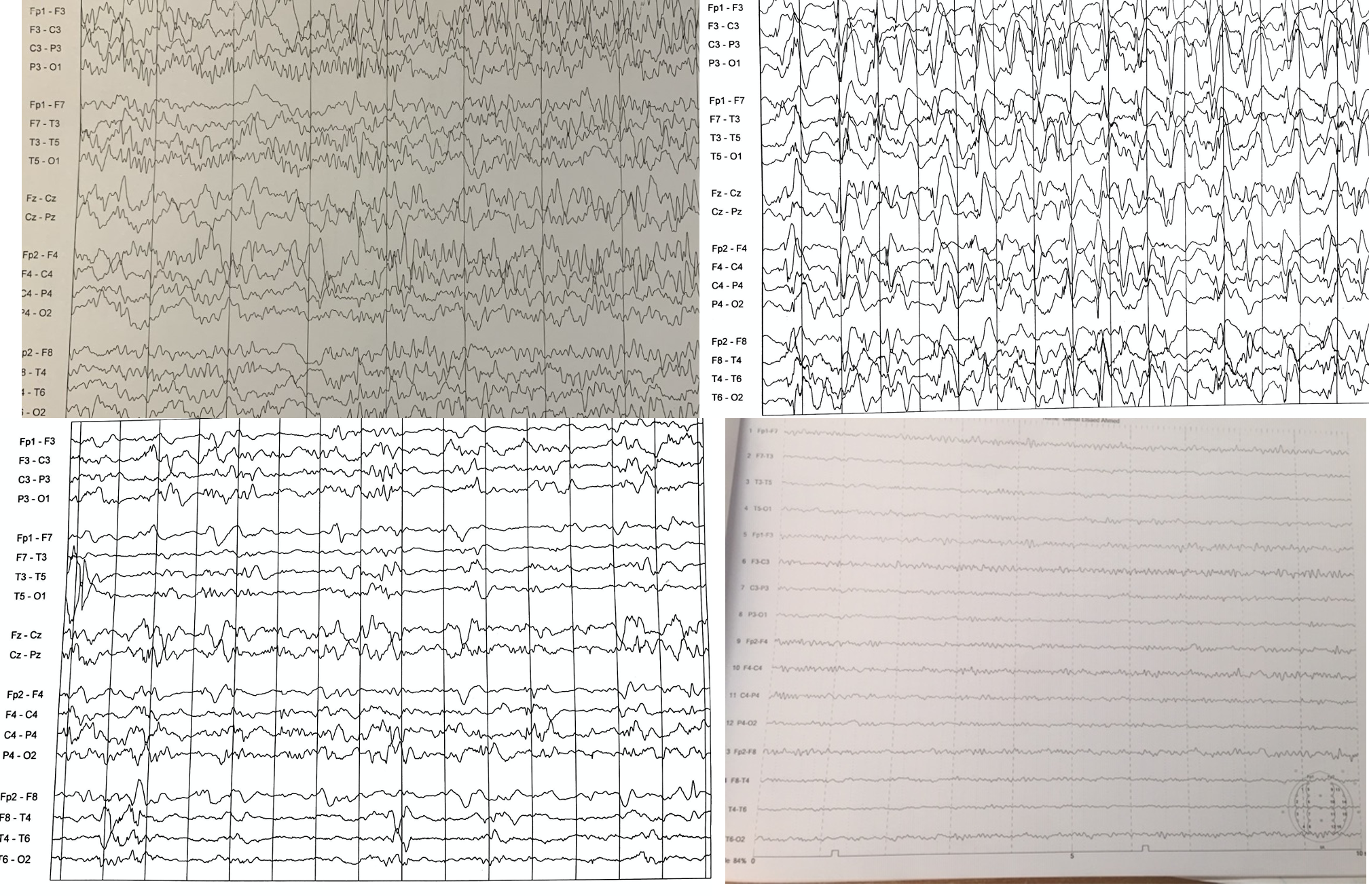
Supplementary Figure 1. EEG recordings** of patient 1 (top left) performed at age 3 years showing pseudo-hypsarrhythmic pattern during wakefulness with runs of 2-3-Hz high amplitude and synchronous slow waves predominant over posterior regions associated with few spikes and slow-wave spikes over temporal regions, patient 2 (top right) done at age 1.5 years showing multifocal epileptic abnormalities within a diffusely slowed high-amplitude during wakefulness and sleep intermixed with a burst-suppression pattern, indicative of active epileptic developmental encephalopathy, patient 3 (bottom left) done at age 3.5 years shows multifocal epileptic abnormalities within a burst-suppression pattern compatible with active epileptic developmental encephalopathy, patient 4 (bottom right) done at age 6 years shows diffusely slowed, low-voltage, monomorphic background activity indicative of subcortical -cortical dysregulation with no evidence of epileptic/paroxysmal abnormalities.


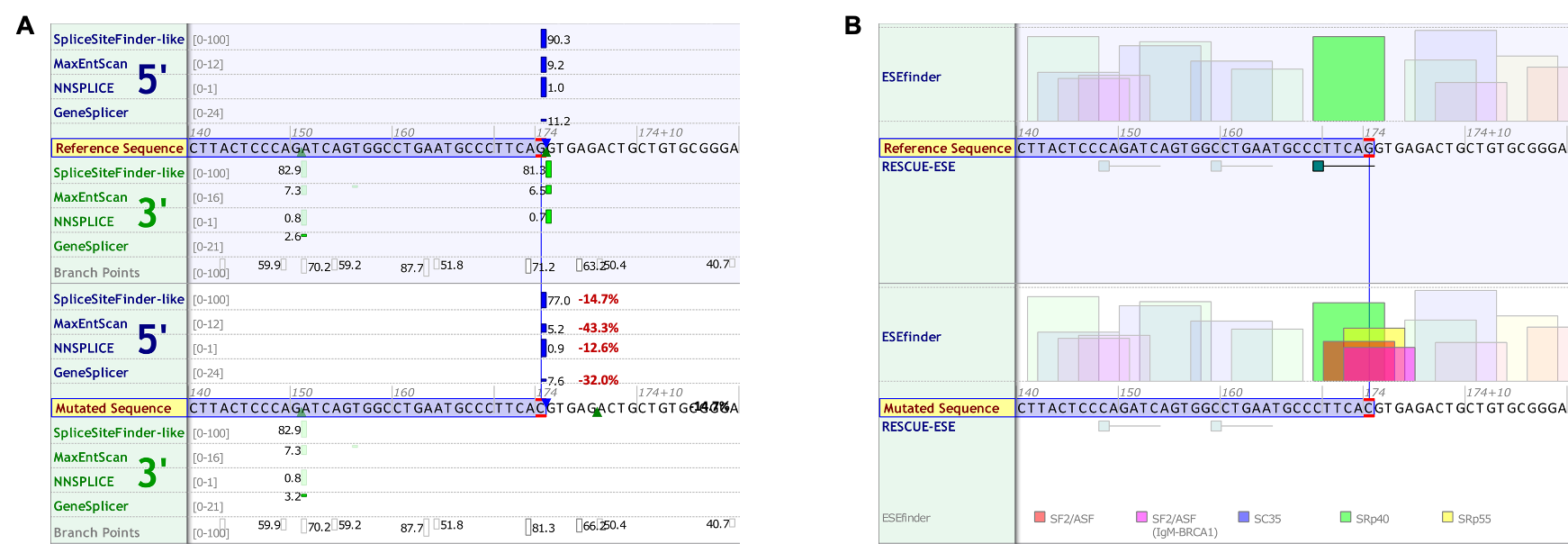


**Supplementary Figure 2. *In silico* splice prediction scores of the *PIGS* c.174G>C variant**. **A.** Four splice prediction tools compare the 5´ and 3´ splice site scores of the wild-type (upper panel) and variant (bottom panel). The native splice donor site is maintained (blue bars), but all splice prediction scores unanimously decrease (lower panel, red). **B.** Analysis with ESEfinder and RESCUE-ESE tools reveal the splicing sequence landscape for the wild-type (upper panel) and variant (bottom panel) sequence. Changes in ESEfinder splicing factor predictions are shown above the sequence in coloured bars. An abolished ESE motif is depicted by a loss of a green box below the sequence. The c.174 nucleotide is outlined in red bars for both (A) and (B).


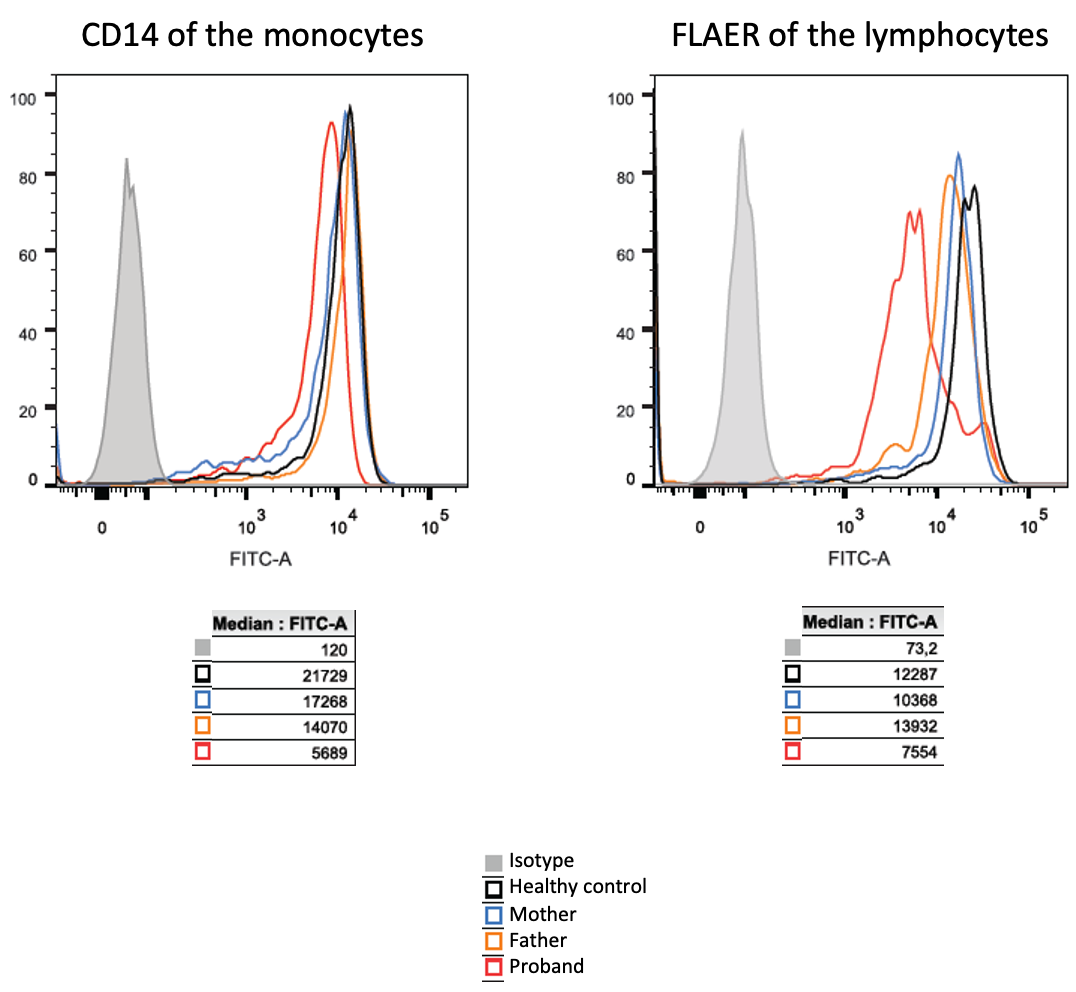


**Supplementary Figure 3. Impact of the *PIGS* variants on individual lymphocyte and monocyte cell-surface GPI-APs.** Flow-cytometry analysis of lymphocytes and monocytes from the same experiments described in Figure 2. Shown is a representative analysis of the amount of cell-surface CD14 on monocytes and FLAER on lymphocytes from triplicate experiments.
